# Retrospective observational analysis of hospital discharge database to characterize primary pulmonary hypertension and its outcomes in Spain from 2004 to 2015

**DOI:** 10.1097/MD.0000000000015518

**Published:** 2019-05-03

**Authors:** Javier de-Miguel-Díez, Ana Lopez-de-Andres, Valentin Hernandez-Barrera, Isabel Jimenez-Trujillo, Manuel Mendez-Bailon, Jose M. de Miguel-Yanes, Nuria Muñoz-Rivas, Martin Romero-Maroto, Rodrigo Jimenez-Garcia

**Affiliations:** a Respiratory Department, Hospital General Universitario Gregorio Marañón, Facultad de Medicina, Universidad Complutense de Madrid (UCM), Instituto de Investigación Sanitaria Gregorio Marañón (IiSGM); b Preventive Medicine and Public Health Teaching and Research Unit, Health Sciences Faculty, Universidad Rey Juan Carlos, Alcorcón; c Internal Medicine Department. Hospital Universitario Clínico San Carlos, Facultad de Medicina, Universidad Complutense de Madrid (UCM), Madrid; d Internal Medicine Department, Hospital General Universitario Gregorio Marañón, Madrid, Facultad de Medicina, Universidad Complutense de Madrid (UCM); e Internal Medicine Department, Hospital Universitario Infanta Leonor, Madrid, Spain.

**Keywords:** comorbidity, cost, hospitalization, incidence, in-hospital mortality, length of hospital stay, primary pulmonary hypertension

## Abstract

To examine trends in the incidence, characteristics and outcomes, and to identify factors associated with in-hospital mortality (IHM) of patients hospitalized for primary pulmonary hypertension (PPH) in Spain (2004–2015).

We included all patients hospitalized with PPH and included in the Spanish National Hospital Discharge Database.

We analyzed 46,883 discharges of patients (7.14% with PPH as their primary diagnosis). Incidence rates decreased from 6.15 cases per 100,000 inhabitants in 2004–06 to 3.40 in 2013–15 (*P* < .001). Mean age rose from 66.43 ± 21.28 to 69.73 ± 21.12 years (*P* < .001) and the proportion of females increased over the study period (58.44% vs 60.71%; *P* < .001). Comorbidity using the Charlson Comorbidity Index (CCI) also increased with 16.07% having CCI ≥3 in 2004–06 vs 21.795 in 2013–15. Median length of hospital stay (LOHS) was 1 day longer in period 2004–06, than in 2013–15 (9 vs 8 days; *P* < .001). The proportion of patients who were considered a readmission and the mean costs increased from 15.7% and 3712.46€ in the first period to 17.14% and 4040.28€ in 2013–15 (*P* < .001). IHM increased from 8.2% in 2004–06 to 9.93% in 2013–15 (*P* < .001). The predictors’ of IHM among PPH patients included comorbidity and use of mechanical ventilation. Primary diagnosis of PPH did not predict higher IHM (OR 1.07; 95%CI 09–1.26).

Our data indicates that the incidence of hospitalizations decreased in Spain between 2004 and 2015. Parallel, LOHS also decreased during this period. By contrast, comorbidity increased over time in PPH patients, as well as readmission rates, costs and IHM.

## Abstract 2

Supplemental Digital Content is available in the text

## 1 Introduction

Pulmonary hypertension (PH) is a complex condition characterized by an abnormal elevation of mean pulmonary artery pressure (equal to or above 25 mmHg), measured during right heart catheterization.^[1]^ Pulmonary arterial hypertension (PAH) is a subcategory of PH that comprises a group of disorders with similar pulmonary vascular pathology.^[2]^ According to the World Health Organization (WHO) classification, PAH is clinically grouped into idiopathic (synonymous with primary), heritable, and PAH with associated conditions such as connective tissue disease, congenital heart disease, HIV infection, and portal hypertension.^[3]^

Primary pulmonary hypertension (PPH) is a rare, progressive, life-threatening condition of unknown etiology.^[4]^ Both its prevalence and its incidence are variable according to different geographical regions, but it is estimated that they are around 15 to 50 per million and 1.0 to 3.3 cases per million population per year, respectively.^[5]^ The natural history of PPH is well established, with a median survival of 2.8 years and estimated 1, 3, and 5 years survival rates of 68%, 48%, and 34%, respectively.^[6]^ The high mortality risk of PPH patients is probably due to various reasons including comorbidities, unique disease phenotypes and a diminished response to PAH targeted therapies.^[7]^ Despite this, treatments have evolved over the past 10 years, resulting in improvements in symptoms, exercise capacity, hemodynamics, time to clinical worsening, and/or survival.^[8]^

The inherent complexity of studying a rare disease makes necessary the creation of registries. These registries are indispensable in the characterization and mapping of the natural history of the disease.^[2]^ However, certain patient populations are less likely referred to reference centers in PH, so the conclusions drawn for registry studies may not be accurate. An alternative approach used in recent years has been to evaluate medical information recorded in administrative databases.^[4]^ We think that research based in hospital discharge diagnoses might be a valid measure of trends in disease prevalence and outcomes for an orphan disease like PPH.

Using the Spanish National Hospital Discharge Database (SNHDD), we aim in this study to: (a) examine trends in the incidence, characteristics and in-hospital outcomes of hospitalizations with PPH (from 2004 to 2015; (b) compare clinical variables among patients according to the diagnosis position of the PPH in the discharge report (primary or secondary); and (c) identify factors associated with in-hospital mortality (IHM) among patients according to the diagnosis position of PPH.

## 2 Methods

### 2.1 Data source

This retrospective observational study was performed using the SNHDD. Details of the characteristics of the SNHDD are available elsewhere.^[9]^ Briefly, this is a nationally representative database, which collect data from the Spanish public hospitals and covers over 95% of hospital admissions in Spain.^[9]^

### 2.2 Patient population

We selected all hospital admissions of patients with a diagnosis of PPH, in the primary or secondary position in their discharge report, and included in the SNHDD database. These hospitalizations were identified using the International Classification of Diseases, Ninth Revision, Clinical Modification (ICD-9-CM) diagnosis code 416.0. A primary diagnosis of PPH refers to a hospitalization, which is direct consequence of PPH, whereas a secondary diagnosis refers to hospitalizations in patients with concomitant PPH who were admitted for other diseases than PPH. PPH was classified as a primary diagnosis if the 416.0 code appear as the first diagnosis position. Otherwise, it was classified as secondary (positions 2–14). We collected data from between January 1, 2004 and December 31, 2015. Those with PPH as primary diagnosis have the disease clearly uncontrolled and those with PPH as secondary diagnosis have the disease more controlled and require hospitalization for other concomitant condition.

### 2.3 Covariates

Clinical characteristics included overall comorbidity assessed by calculating the CCI at the time of discharge.^[10,11]^ The index includes 17 disease categories that are counted so an overall score is obtained for patient. We divided patients into 3 categories according to the number of disease (none; 1 or 2 and 3 or more).

We also identified the following in-hospital procedures using the ICD-9-CM codes: respiratory function test (89.37), lung gammagraphy (92.15), ultrasound of lower limbs (88.77), right heart catheterization (37.21), computed tomography of the chest (87.41), pulmonary arteriography (88.43), invasive mechanical ventilation (96.04), Swan-Ganz catheter (89.64), heart echocardiogram (88.72), and lung transplant (33.50, 33.51, 33.52, and 33.56).

We considered a readmission those patients who had been discharged in the previous month from the same hospital days), and the median of LOHS.

Costs were calculated using diagnosis-related groups for the disease.^[12]^ Costs analyzed were inflation adjusted.

### 2.4 End points

The end points of our investigation were the incidence of hospitalizations and IHM. In addition, we assessed trends in patients admitted with primary and secondary diagnosis of PPH separately. IHM was defined as the proportion of patients who died during admission for each year of study.

### 2.5 Statistical analysis

We considered 4 time periods 2004–06; 2007–09; 2010–12 and 2013–15). We calculated incidence rates by dividing the number of cases of PPH per year, sex, and age group by the corresponding number of people in that population group. The data necessary for these calculations were obtained from the Spanish National Institute of Statistics.^[13]^ The incidence rates were expressed per 100,000 inhabitants. Poisson regression models were used to assess trends in the incidences.

A descriptive statistical for continuous variables was done using means with standard deviations or medians with interquartile ranges (LOHS). Categorical variables are expressed as proportions. To assess association between variables we used χ^2^ test for linear trend (proportions), ANOVA (means), and Kruskall-Wallis (medians).

We performed 3 multivariable logistic regression analyses to identify variables associated with IHM, one for each diagnosis position of PPH (primary, secondary, both). In these 3 models, we checked for interactions, we limited interactions to first order (2 by 2), and none of them showed a significant association. Estimates were OR with their 95%CI.

Stata version 10.1 was used for statistical analysis (Stata, College Station, TX). Statistical significance was set at *P* < .05 (2-tailed).

### 2.6 Ethical aspects

The study maintains data confidentiality at all times. Given the anonymous and mandatory nature of the database, it was not necessary to obtain informed consent or approval by an ethics committee in accordance with Spanish legislation.

## 3 Results

The total number of patients hospitalized in Spain between 2004 and 2015 with a diagnosis of PPH in their discharge report was 46,883. Patients with a PPH as their primary diagnosis accounted for 7.14% of the total.

### 3.1 Trends in primary pulmonary hospitalizations and in-hospital outcomes

We found that the incidence of PPH decreased significantly from 6.15 in 2002–06 to 3.40 cases per 100,000 inhabitants in 2013–15 (*P* < .001). Mean age increased significantly over time (66.43 ± 21.28 years in 2004–06 vs 69.73 ± 21.12 years in 2013–15; *P* < .001). Analysis of sex distribution showed an increase in the proportion of women over the study period (58.44% vs 60.71%; *P* < .001) (Table 1).

**Table 1:**
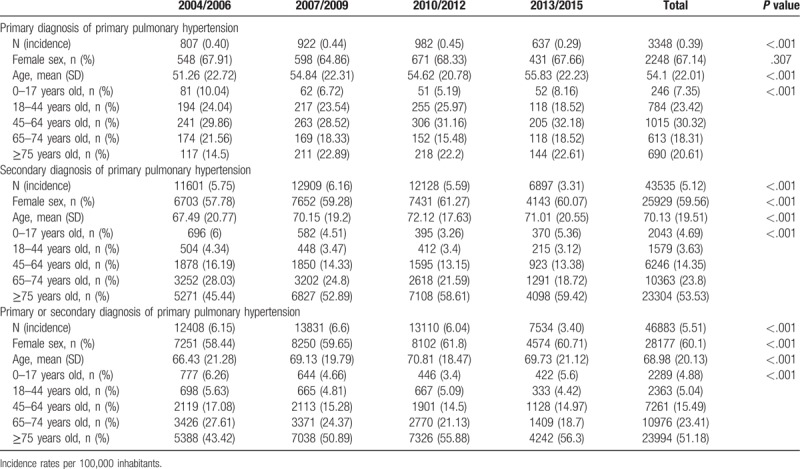
Incidence rates and distribution according to age and sex for primary pulmonary hypertension as primary or secondary diagnosis in Spain from 2004 to 2015.

Incidence rates in PPH as primary and secondary diagnosis also decreased over time (0.40 and 5.75 cases per 100,000 inhabitants in 2004–06, respectively vs 0.29 and 3.31 in 2013–15, respectively). As can been seen in Table 1, the mean age increased over time in both primary and secondary diagnosis of PPH. Remarkably, over the entire time period, the patients admitted with PPH as the primary diagnosis were younger than those with a secondary diagnosis of PPH (54.1 ± 22.01 years vs 70.13 ± 19.51 years; *P* < .001) and were female in a significantly higher proportion (67.14% vs 59.56%; *P* < .001).

We detected a significant increase in comorbidity using CCI ≥3 over time (16.07% in 2004–06 vs 21.795 in 2013–15) in PPH admissions in any diagnostic position. Median LOHS for admissions for PPH was 9 days in period 2004–06, decreasing to 8 days in 2013–15 (*P* < .001). The proportion of patients considered a readmission and mean costs also increased significantly during the study period in the bivariable analysis, from 15.7% and 3712.46€ in the period 2004–06 to 17.14% and 4040.28€ in 2013–15 (Table 2).

**Table 2:**
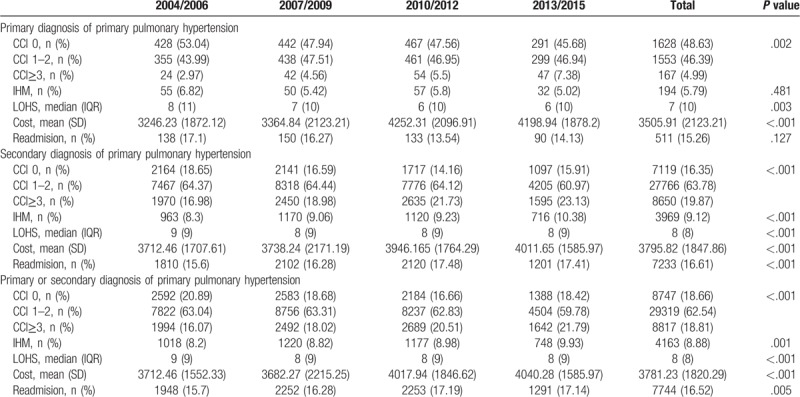
Charlson comorbidity index (CCI), length of hospital stay (LOHS), cost, in-hospital mortality (IHM) and readmissions for patients hospitalized for primary pulmonary hypertension as primary or secondary diagnosis in Spain from 2004 to 2015.

For the total time period in PPH hospitalizations as primary and secondary diagnosis, crude IHM was 8.88%. IHM increased significantly (*P* < .001) over time from 8.2% in 2004–06 to 9.93% in 2013–15. However, in PPH admission coded as primary diagnosis, the crude IHM decreased slightly but not significantly from 6.82% to 5.02% over the study period (*P* = .481) (Table 2).

As can been seen in Table 2 from 2004 to 2015, the patients admitted with PPH as the primary diagnosis had significant lower CCI (≥3, 4.99% vs 19.87%), LOHS (median 7 days vs 8 days), readmission (15.26% vs 16.61%), costs (mean 3505.91€ vs 3795.82€) and IHM (5.79% vs 9.12%) than in those with PPH as a secondary diagnosis,

The most common associated comorbidities for patients hospitalized for PPH in any diagnostic position were congestive heart failure (50.76%), chronic obstructive pulmonary disease (COPD) (26.35%), and diabetes not complicated (23.63%). Patients with a primary diagnosis of PPH had lower specific clinical conditions than those with a secondary diagnosis. However, the prevalence of rheumatoid disease, mild liver disease and AIDS was higher, as can been seen in Table 3.

**Table 3:**
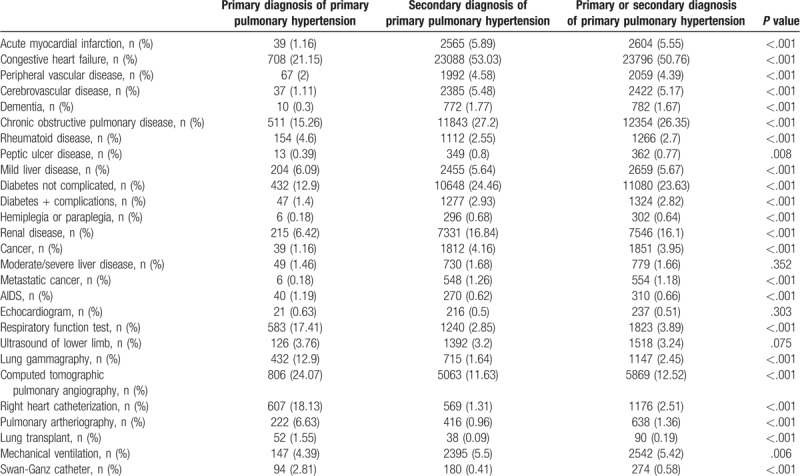
Associated comorbidities included in the Charlson Comorbidity Index and diagnosis procedures and treatments for patients hospitalized for primary pulmonary hypertension as primary or secondary diagnosis in Spain from 2004 to 2015.

Regarding specific diagnosis procedures and treatments for patients hospitalized for PPH, prevalence of all procedures and treatments was higher in patients with a primary diagnosis of PPH, except invasive mechanical ventilation (4.39% vs 5.5%; *P* = .006) (Table 3).

The most common concomitant conditions for patients with PPH coded as the secondary diagnosis was congestive heart failure (24.0%), other diseases of lung (7.3%) and chronic bronchitis (4.8%) (Supplementary Table 1).

### 3.2 Factors associated with in-hospital mortality

The factors independently associated with IHM according to diagnosis position of PPH are shown in Table 4. Males had a lower risk of dying during their hospitalization than females after multivariable adjustment only in secondary diagnosis of PPH (Odds Ratio, OR 0.93; 95%CI 0.86–0.99). Those patients with a secondary diagnosis of PPH with higher age (≥75 years) have significantly higher OR (1.57; 95%CI 1.27–1.95) for IHM than those with 18 to 44 years old group. Comorbidities according to the CCI were significant risk factors for IHM in the 3 populations analyzed.

**Table 4:**
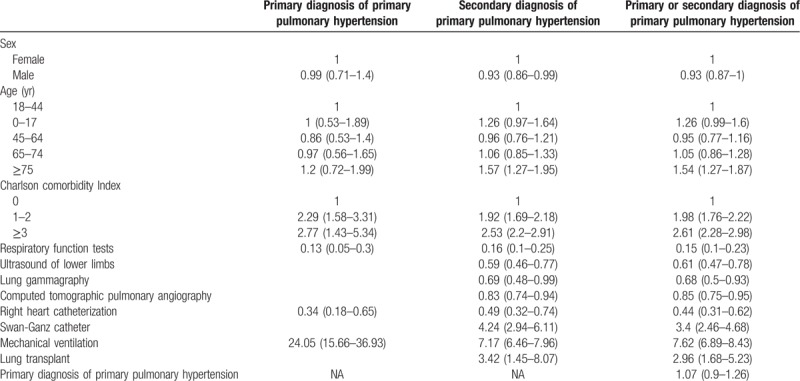
Factors associated with in-hospital deaths among patients hospitalized for primary pulmonary hypertension as primary or secondary diagnosis in Spain from 2004 to 2015.

Invasive mechanical ventilation increases the risk of dying in the hospital in patients with PPH as primary and secondary diagnosis. In the 3 groups studied, respiratory function tests and right heart catheterization were associated with a lower risk of dying.

In patients with secondary diagnosis of PPH, IHM was significantly higher in those with Swan-Ganz catheter (OR 4.24; 95%CI 2.94–6.11) and in those with lung transplant (OR 3.42; 95%CI 1.45–8.07). However, factors associated with lower IHM were ultrasound of lower limbs, lung gammagraphy and computed tomographic pulmonary angiography (Table 4).

Finally, primary diagnosis of PPH did not predict IHM (OR 1.07; 95%CI 0.9–1.26).

## 4 Discussion

In this study, we have found a significant decrease in the incidence of hospitalizations by PPH in Spain between 2004 and 2015, regardless if PPH was coded as primary or secondary diagnosis. Advances in the management of PPH have led to the approval of new therapies in last 15 years. As a result, clinical practical guidelines have tailored such treatments either as single drug therapy or in combination, which may have influenced the results of our study.^[14]^ A decline in PPH discharge diagnoses was also reported by Link et al^[4]^ from 2001 and onward. All this occurred during years where total PH discharge diagnoses increased.^[15]^ This data would indicate that the rise in the number of hospitalizations due to PH can be attributed to an increase in hospital admissions due to secondary causes.^[16]^

There seem to be 2 populations susceptible to have PPH that is young patients and the elderly ones. However, contemporary reports from PH registries show a new demographic picture for the PPH population.^[17,18]^ Therefore, prevalence among the elderly is increasing, with a mean age of 50 to 65 years reported at diagnosis,^[19–22]^ but the reason for this shift is not clear.^[23]^ In relation to gender, epidemiological studies have reported an increased prevalence of PAH among women, while male PAH patients seem experience worse outcomes.^[24]^ We also found an increase in mean age and in the percentage of women over the study period when we analyzed all the patients, but we did not observe changes in the percentage of women when PPH was coded as primary diagnosis. In addition, our analysis revealed that patients admitted with PPH as the primary diagnosis were younger than those with a secondary diagnosis and were female in a significantly higher proportion.

Data relating comorbidity to outcome in PPH patients are scarce.^[16,21,25]^ We observed a significant increase in comorbidity over time in PPH admission in any diagnostic position. However, patients admitted with PPH as the primary diagnosis had significant lower CCI. The most common associated comorbidities for patients hospitalized for PPH in any diagnosis position were congestive heart failure and COPD.

Concerning the diagnostic tests, the rates of echocardiogram and right catheterization found in the study were pretty low. However, we included patients hospitalized with PPH but the diagnosis could have been made previously, which would justify these results.

While the admission rates for PPH decreased, the overall cost per hospitalization increased over the study period in our analysis. Similar results were found by Anand et al.^[26]^ They speculated that rise in costs is related to the increased acuity of illness in those patients with PAH who are hospitalized; thus, as outpatient care has improved, patient admitted to the hospital are generally more sick. This assertion is supported by the reported increased prevalence of significant comorbidities, as it happened in our study. On the other hand, the advance in the therapeutic armamentarium for this disease and the increasing use of combination therapy throughout the study period may have also contributed to the increase in costs.^[27]^ However, the contribution of medication costs to overall hospitalization costs were not registered in our study.

We observed a significant decrease in LOHS and an increase in hospital readmissions in PPH patients over the study period. The exact reason for the decrease in LOHS is unknown. However, it may be attributed to advances in the outpatient diagnostic and treatment modalities over the analyzed time period. By contrast, other authors have noted an increase in LOHS in hospitalized patients with PH during a similar time period of time, although this may seem counterintuitive.^[16]^ Excessively early discharge in our study could have leaded to an increasing rate of readmissions. Burke et al also found a high percentage of PAH patients who required readmission after discharge. In addition, the costs of readmissions was significantly higher and it was associated with increased mortality.^[28]^

Mortality remains high despite improvements in the PH treatment in the past 2 decades.^[29]^ We found and increase in PPH mortality rates between 2004 and 2015. However, in PPH admissions coded as primary diagnosis, the IHM decreased slightly but not significantly, over the study period. In the same way, Link et al^[4]^ reported a decline in PPH mortality from 2003 and onward. Among factors independently associated with IHM in all population analyzed in our study were comorbidity and use of mechanical ventilation. In addition, males had a lower risk of dying during their hospitalization than females only in secondary diagnosis of PPH. Furthermore, patients with a secondary diagnosis of PPH with advanced age (≥75 years) have a higher risk of IHM than those with 18 to 44 years. Other established prognostic factors in PPH patients are functional class III to IV and the distance walked in 6 minutes, but we did not have information of them in our study.^[30,31]^

In patients with secondary diagnosis of PPH, IHM was significantly higher in those with lung transplant. Previous studies have identified PH as a risk factor of mortality after lung transplantation.^[32]^ However, factors associated with lower IHM in our study were ultrasound of lower limbs, lung gammagraphy and computed tomographic pulmonary angiography. Increased use of imaging modalities would help to establish an early diagnosis and, thus contribute to a reduced mortality.^[16]^ On the other hand, primary diagnosis of PPH did not predict IHM in our analysis.

The more precise diagnostic of chronic pulmonary embolism is another possible reason for the reduction of incidence of primary PH. Unfortunately, the code for chronic pulmonary embolism was introduced in the SNHDD in year 2012 so it is not possible to assess time trend before this year. As can be seen in Supplementary Table 2 the trend in the number of primary and secondary diagnosis of this condition shows a significant increment in the number of cases; mainly as secondary diagnosis, from 2012 to 2015. However, more studies are needed to confirm this trend.

### 4.1 Limitations

The limitations of our investigation should be mentioned. First, admissions are identified by and ICD-9 code and not by the WHO classification of PH.^[3]^ However, the code used to identify patients with PPH is more specific than codes used to identify patients with secondary PH. In any case we think, like other authors, that the methodology used is reliable to study the magnitude characteristics and consequences of PH in the hospitalizations.^[15,16,30,33,34]^ Second, we miss relevant information, such as PHH duration or medications, laboratory results, physiological measurements (BP, BMI, waist circumference), lifestyles or family history of diseases and, therefore cannot be analyzed, because are not collected by the SNHDD. Nonetheless, we provide data from an entire country over a 12 year period using a database that includes data from over 95% of hospitalizations.

### 4.2 Future directions

This is an epidemiological study, and as commented before we lack relevant data given the characteristics of the database used, it is not possible or intended to modify the clinical practice. Further investigations are required to identify the causes responsible for these findings.

## 5 Conclusion

In conclusion, this analysis of a large, population-based cohort of PPH patients, indicates that the incidence of hospitalizations decreased in Spain between 2004 and 2015. Parallel, LOHS also decreased during this period. By contrast, increased comorbidity over time in PPH patients, as well as readmission rates, costs and HMI.

## Author contributions

**Conceptualization:** Javier de Miguel-Diez, Nuria Muñoz-Rivas, Rodrigo Jimenez-Garcia.

**Formal analysis:** Valentin Hernandez-Barrera, Isabel Jimenez-Trujillo.

**Funding acquisition:** Javier de Miguel-Diez.

**Investigation:** Rodrigo Jimenez-Garcia.

**Methodology:** Javier de Miguel-Diez, Ana Lopez-de-Andres, Valentin Hernandez-Barrera, Isabel Jimenez-Trujillo, Manuel Mendez-Bailon, Jose M de Miguel-Yanez, Nuria Muñoz-Rivas, Martin Romero-Maroto, Rodrigo Jimenez-Garcia.

**Supervision:** Ana Lopez-de-Andres, Rodrigo Jimenez-Garcia.

**Writing – original draft:** Javier de Miguel-Diez.

**Writing – review & editing:** Ana Lopez-de-Andres, Manuel Mendez-Bailon, Jose M de Miguel-Yanez, Nuria Muñoz-Rivas, Martin Romero-Maroto, Rodrigo Jimenez-Garcia.

## Supplementary Material

Supplemental Digital Content
