# Supplementary material for: Retrospective observational analysis of hospital discharge database to characterize primary pulmonary hypertension and its outcomes in Spain from 2004 to 2015
Source: Medicine (Baltimore). 2019 May 3;98(18):e15518. doi: 10.1097/MD.0000000000015518 (PMC6504269; doi:10.1097/MD.0000000000015518)
Supplement: Supplemental Digital Content [file medi-98-e15518-s001.doc]

**Supplementary Table 1.** Most common primary diagnosis in patients discharged with a primary pulmonary hypertension in a secondary diagnosis position.

| Diagnosis | Code | N | % |
| --- | --- | --- | --- |
| Congestive heart failure, unspecified | 428 | 10461 | 24.0 |
| Other diseases of lung | 518 | 3159 | 7.3 |
| Chronic bronchitis | 491 | 2085 | 4.8 |
| Cardiac dysrhythmias | 427 | 1661 | 3.8 |
| Hypertensive heart disease | 402 | 1251 | 2.9 |
| Pneumonia, unspecified organism | 486 | 1153 | 2.6 |
| Other respiratory disease | 519 | 1074 | 2.5 |
| Acute pulmonary heart disease | 415 | 1038 | 2.4 |
| Acute myocardial infarction | 410 | 1004 | 2.3 |
| Other diseases of endocardium | 424 | 840 | 1.9 |
| Bulbus cordis anomalies and anomalies of cardiac septal closure | 745 | 631 | 1.4 |
| Acute bronchitis and bronchiolitis | 466 | 620 | 1.4 |
| Occlusion of cerebral arteries | 434 | 603 | 1.4 |
| Symptoms involving respiratory system and other chest symptoms | 786 | 526 | 1.2 |
| Other acute and subacute forms of ischemic heart disease | 411 | 525 | 1.2 |
| Diseases of mitral valve | 394 | 514 | 1.2 |
| General symptoms | 780 | 485 | 1.1 |
| Diseases of mitral and aortic valves | 396 | 472 | 1.1 |
| Complications peculiar to certain specified procedures | 996 | 436 | 1.0 |
| Other forms of chronic ischemic heart disease | 414 | 419 | 1.0 |
| Other rheumatic heart disease | 398 | 417 | 1.0 |

Supplementary table 2. Trend in the hospitalizations with a primary or secondary diagnosis of chronic pulmonary embolism (ICD-9-CM diagnosis code: 416.2) from 2012 to 2015 in Spain

|  |  | Men | | | | | Women | | | | | Both |
| --- | --- | --- | --- | --- | --- | --- | --- | --- | --- | --- | --- | --- |
|  | Year | 15-44 years | 45-64 years | 65-74 years | 75+ years | TOTAL | 15-44 years | 45-64 years | 65-74 years | 75+ years | TOTAL | TOTAL |
| Primary | 2012 | 13 | 13 | 13 | 22 | 61 | 8 | 19 | 22 | 39 | 88 | 149 |
|  | 2013 | 8 | 32 | 25 | 33 | 98 | 6 | 21 | 21 | 46 | 94 | 192 |
|  | 2014 | 12 | 27 | 14 | 27 | 80 | 10 | 20 | 19 | 57 | 106 | 186 |
|  | 2015 | 8 | 23 | 16 | 26 | 73 | 9 | 26 | 28 | 52 | 115 | 188 |
|  | Total | 41 | 95 | 68 | 108 | 312 | 33 | 86 | 90 | 194 | 403 | 715 |
| Secondary | 2012 | 25 | 106 | 99 | 220 | 450 | 17 | 77 | 96 | 335 | 525 | 975 |
|  | 2013 | 32 | 125 | 122 | 302 | 583 | 20 | 99 | 103 | 395 | 619 | 1202 |
|  | 2014 | 26 | 134 | 145 | 322 | 627 | 39 | 111 | 135 | 440 | 725 | 1352 |
|  | 2015 | 29 | 164 | 177 | 396 | 766 | 35 | 122 | 126 | 524 | 807 | 1573 |
|  | Total | 112 | 529 | 543 | 1240 | 2426 | 111 | 409 | 460 | 1694 | 2.676 | 5102 |
| Both | Total | 153 | 624 | 611 | 1348 | 2738 | 144 | 495 | 550 | 1888 | 3079 | 5817 |

No cases were found under the age of 15
